# Open or Close the Gate – Stomata Action Under the Control of Phytohormones in Drought Stress Conditions

**DOI:** 10.3389/fpls.2013.00138

**Published:** 2013-05-13

**Authors:** Agata Daszkowska-Golec, Iwona Szarejko

**Affiliations:** ^1^Department of Genetics, Faculty of Biology and Environmental Protection, University of SilesiaKatowice, Poland

**Keywords:** stomata, guard cells, phytohormones, abiotic stress, ABA, jasmonic acid, crosstalk

## Abstract

Two highly specialized cells, the guard cells that surround the stomatal pore, are able to integrate environmental and endogenous signals in order to control the stomatal aperture and thereby the gas exchange. The uptake of CO_2_ is associated with a loss of water by leaves. Control of the size of the stomatal aperture optimizes the efficiency of water use through dynamic changes in the turgor of the guard cells. The opening and closing of stomata is regulated by the integration of environmental signals and endogenous hormonal stimuli. The various different factors to which the guard cells respond translates into the complexity of the network of signaling pathways that control stomatal movements. The perception of an abiotic stress triggers the activation of signal transduction cascades that interact with or are activated by phytohormones. Among these, abscisic acid (ABA), is the best-known stress hormone that closes the stomata, although other phytohormones, such as jasmonic acid, brassinosteroids, cytokinins, or ethylene are also involved in the stomatal response to stresses. As a part of the drought response, ABA may interact with jasmonic acid and nitric oxide in order to stimulate stomatal closure. In addition, the regulation of gene expression in response to ABA involves genes that are related to ethylene, cytokinins, and auxin signaling. In this paper, recent findings on phytohormone crosstalk, changes in signaling pathways including the expression of specific genes and their impact on modulating stress response through the closing or opening of stomata, together with the highlights of gaps that need to be elucidated in the signaling network of stomatal regulation, are reviewed.

## Introduction

Stomata are specialized epidermal structures that are essential for plant survival and productivity. These structures consist of two guard cells around a pore. Every stoma is a molecular valve that acts in gas exchange, mainly CO_2_ and O_2_, which is necessary for optimal photosynthesis and which restricts water loss by modulating the transpiration level. The genes that are involved in the process of stomata development were crucial for the movement of plants from water to land during evolution since stomata facilitated gas exchange while limiting desiccation. The stomatal morphogenesis pathway has been identified in detail in *Arabidopsis thaliana* through investigations of many mutants with an impaired stomatal pattern or with other morphological defects in their epidermal cells. Cell distribution and differentiation require a balance between proliferation and cell specification in time and space. The differentiation of stomata is preceded by at least one asymmetric as well as a few symmetric cell divisions. It requires three different types of precursor cells: the meristemoid mother cell (MMC), meristemoids and the guard mother cell (GMC). The last step of stomatal development is the differentiation of the stoma itself within the structure of the guard cells. The number and pattern of stomata varies in different organs in *A. thaliana*. A common feature of patterning is that stomata are separated from each other by at least one epidermal cell. This pattern ensures the presence of neighbor cells for ion exchange, which is necessary for the regulation of the aperture width. For this reason, neighbor cells are part of a stomatal complex (Nadeau and Sack, 2002; Nadeau, 2009; Lau and Bergmann, 2012; Pillitteri and Torii, 2012; Vatén and Bergmann, 2012). Recent research has shown that the mode of action of stomata depends on the integration of environmental and intracellular signals. Many environmental factors such as CO_2_ concentration, biotic and abiotic stresses, and additionally different plant hormones, can modulate stomatal reaction. For plants that encounter dehydration stress, the most essential factor is the ability of stomata to close and thus prevent excess water loss. Opening and closing is achieved by the swelling and shrinking of the guard cells, which is driven by ion exchange; cytoskeleton reorganization and metabolite production; the modulation of gene expression and the posttranslational modification of proteins (reviewed in Kim et al., 2010). Swelling of the guard cells results in stomata opening since the content of ions and osmolites within them makes them bigger and thus able to move away from each other making the stomatal aperture larger. In contrast, closing is an opposite mechanism and results in the shrinking of the guard cells when the efflux of ions occurs.

Stomatal closure is the earliest plant response to water deficit (Schroeder et al., 2001b). This rapid reaction is regulated by a complex network of signaling pathways, in which the major and the best-known player, abscisic acid (ABA), acts in concert with jasmonates (JA), ethylene, auxins, and cytokinins (Nemhauser et al., 2006; Huang et al., 2008). The complexity of the response is mainly dependent on the initial threshold of stress and individual plant’s stress history. Generally, ABA and JA are positive regulators of stomatal closure, while auxin and cytokinins are positive regulators of stomatal opening. The mode of action of ethylene is ambiguous because it can act as a positive or negative regulator, depending on the tissue and conditions (Nemhauser et al., 2006; Huang et al., 2008).

This paper presents a comprehensive review of the genetic and molecular basis of stomata action under the control of phytohormones, particularly when response to drought stress is considered.

## Open or Close the Gate – The Role of ABA, Ion Channels, and Diurnal Cycle in Stomatal Movements Regulation

### The regulatory role of ion channels localized in the guard cell membrane in the opening and closing stomata

**Figure 1:**
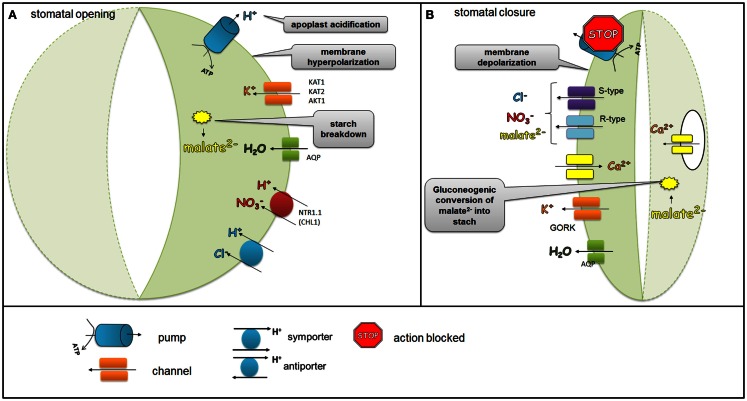
**Regulation of ion channels, pumps, and transporters localized in the plasma membrane of the guard cells during stomatal opening and closure**. During stomatal opening **(A)** H^+^-ATPase pumps H^+^ from the guard cells and hyperpolarizes the membrane, which leads to the activation of K^+^ inward rectifying channels (KAT1, KAT2, AKT1). Anionic species such as malate^2−^ from the breakdown of starch and transported ${\text{NO}}_{3}^{-}$ and Cl^−^ ions contribute to the intracellular solute buildup that can mediate the import of sugars or can be used for the synthesis of sugars. Ions supplied into the guard cells together with water transported via aquaporins generate the turgor that is needed to keep stomata opened. During stomatal closure **(B)**, H^+^-ATPase is inhibited and S-type and R-type anion channels are activated. As the plasma membrane is depolarized, S-type and R-type channels facilitate the efflux of malate^2−^, Cl^−^, and ${\text{NO}}_{3}^{-}$. At the same time, K^+^ outwardly rectifying channels such as GORK are activated through the depolarization of the membrane, which leads to the efflux of K^+^. The decreased level of malate^2−^ is also caused by the gluconeogenic conversion of malate into starch. The elevation of the Ca^2+^ concentration as a result of the release of Ca^2+^- via channels situated in both the plasma membrane and in the tonoplast is another event that accompanies stomatal closure.

The guard cell turgor is dynamically adjusted to environmental conditions and hormonal signals in order to facilitate the proper gas exchange and prevent excessive water loss. Mature guard cells do not have plasmodesmata and for this reason most influx and efflux of solutes occurs via ion channels, transporters, and pumps that are localized in the plasma membrane (PM). The action of ion channels, transporters, and pumps that are essential for stomatal function is well documented and supported by molecular studies involving mutants in the genes encoding these protein. During the opening of the stomata, the H^+^-ATPase pump mediates the efflux of H^+^ from the guard cells. In plants, H^+^-ATPases belong to the multi-gene family of the P-type ATPases, with 11 genes in *Arabidopsis*, which are all expressed in the guard cells (Ueno et al., 2005). In the guard cells, the action of H^+^-ATPase activity is positively regulated by blue light and auxins, whereas Ca^2+^ and ABA act as negative regulators. The efflux of H^+^ hyperpolarizes the PM and leads to K^+^ uptake via activation of inward K^+^ rectifying channels, such as KAT1 (potassium channel in *Arabidopsis thaliana* 1), KAT2 (potassium channel in *Arabidopsis thaliana* 2), and AKT1 (*Arabidopsis thaliana* K^+^ transporter 1) (Schachtman et al., 1992; Pilot et al., 2001; Szyroki et al., 2001). Another signal that activates the influx of K^+^ via K^+^ channels is the acidification of the apoplast as a result of H^+^ extrusion from the guard cells. K^+^ uptake is balanced by counter-ions, mainly Cl^−^ obtained from the apoplast, malate^2−^ that is derived from starch breakdown or ${\text{NO}}_{3}^{-}$. The last one is transported from the apoplast by a nitrate transporter AtNRT1.1 (CHL1) [nitrate transporter 1 (chlorina1)]. The importance of ${\text{NO}}_{3}^{-}$ uptake was confirmed by an analysis of an *Arabidopsis* *clh1* mutant. The stomatal apertures of the *chl1* mutant were smaller than those of the wild-type when nitrate was supplied. Furthermore, the *chl1* mutant was drought tolerant (Guo et al., 2003). Ions supplied into the guard cells together with water transported via aquaporins generate the turgor that are necessary to keep stomata open (Figure 1A).

During stomatal closure, the inhibition of H^+^-ATPase and the activation of anion channels together result in membrane depolarization. Anion channels such as rapid channels (R-type) and slow channels (S-type) facilitate the efflux of malate^2−^, Cl^−^, and ${\text{NO}}_{3}^{-}$ (Roelfsema et al., 2004; Roelfsema and Hedrich, 2005). The decreased level of malate^2−^ in guard cells is also linked with the gluconeogenic conversion of malate^2−^ into starch (Willmer and Fricker, 1996). Membrane depolarization creates a driving force for the efflux of K^+^ via K^+^ outwardly rectifying channels such as GORK (guard cell outwardly rectifying K^+^ channel) (Jeanguenin et al., 2008). An *Arabidopsis* *gork* mutant displayed impaired stomatal closure, thus confirming the important role of GORK in elimination K^+^ ions and in the facilitation of stomatal closure (Hosy et al., 2003). Another event that accompanies stomatal closure is an elevation of the cytoplasmic Ca^2+^ concentration as a result of Ca^2+^-release via channels situated in both the PM and in the tonoplast (MacRobbie, 2006). Ca^2+^ channels are encoded by genes from three gene-families: TPC1 (two-pore channel 1) (Peiter et al., 2005), CNGC (cyclic nucleotide gated channel) (Finn et al., 1996), and GLR (glutamate receptor) (Lacombe et al., 2001). Taken together, the efflux of solutes from the guard cells leads to a reduced turgor and stomatal closure (Figure 1B).

### Abscisic acid – how the proper level of the main regulator of stomatal movements is achieved in plants

Abscisic acid has been postulated as a main regulator of stomatal movements but its proper functioning depends on the appropriate level of biologically active ABA within the plant cells. This is achieved by synchronized processes such as ABA biosynthesis, catabolism, conjugation/deconjugation, and transport. These processes, which are well recognized and studied in various species, have confirmed the function of many enzymes involved in the biosynthesis, catabolism, conjugation/deconjugation, and transport of ABA. The exception, not fully recognized yet, is ABA signal transduction pathway. Although ABA has been the focus of many research groups since the early 90s, there are still many questions in regards to the function of the proteins involved in ABA signaling, protein interactions or the impact of the components of signalosome on specific physiological responses. Therefore, with the progress in studies on ABA signaling, the state of knowledge and the already known interaction web should be updated and verified.

**Figure 2:**
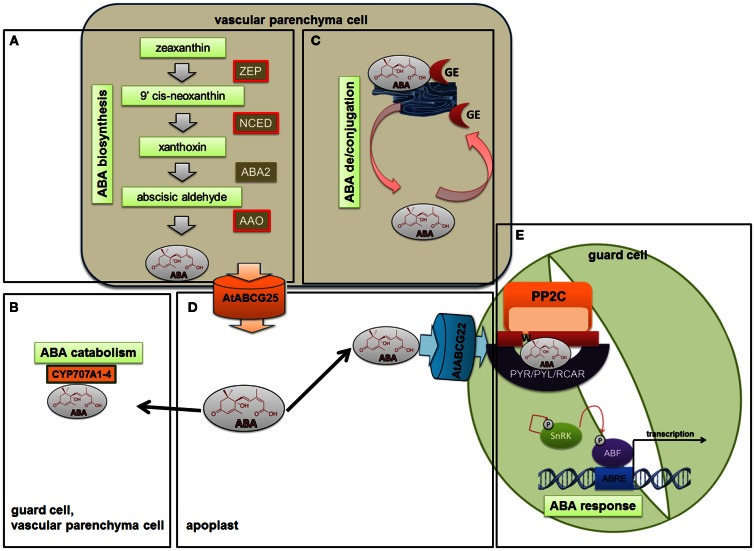
**Abscisic acid biosynthesis, catabolism, deconjugation, transport, and signaling**. ABA biosynthesis **(A)** is mainly induced by upregulating *NCED3*, *ZEP*, and *AAO* genes. At the same time as the biosynthesis of ABA is induced, the catabolism **(B)** that is performed by CYP707A1-4 is inhibited. The balance between active and inactive ABA in the cell is achieved not only by the regulation of biosynthesis and catabolism but also by ABA conjugation and deconjugation. The most widespread conjugate is the ABA glucosyl ester (ABA-GE), which is catalyzed by ABA glucosyltransferase **(C)**. ABA delivery to the guard cells via ABCG transporters such as AGCG22 **(D)** promotes a cascade of reactions. The core of early ABA signaling involves ABA receptors – PYR/PYL/RCAR proteins, PP2Cs, and SnRKs **(E)**. After binding ABA to the receptor, the negative regulatory action of PP2Cs is inhibited and SnRKs are able to phosphorylate and activate downstream targets in order to transduce the ABA signal.

Abscisic acid is synthesized in the plastids and cytosol, mainly in the vascular parenchyma cells but also in the guard cells, through the cleavage of a C40 carotenoid precursor, followed by a two-step conversion of the intermediate xanthoxin into ABA via ABA-aldehyde (Taylor et al., 2000; Finkelstein and Rock, 2002; Schwartz et al., 2003; Endo et al., 2008; Melhorn et al., 2008). The pathway begins with isopentenyl pyrophosphate (IPP), which is the biological isoprene unit and the precursor of all terpenoids, as well as many plant hormones. The next step is the epoxidation of zeaxanthin and antheraxanthin into violaxanthin, which is then catalyzed by zeaxanthin epoxidase (ZEP) (Marin et al., 1996). After a series of violaxanthin modifications that are controlled by the enzyme ABA4, violaxanthin is converted into 9-cis-epoxycarotenoid (North et al., 2007). Oxidative cleavage of the major epoxycarotenoid 9-cis-neoxanthin by the 9-cis-epoxycarotenoid dioxygenase (NCED) yields a C15 intermediate – xanthoxin (Schwartz et al., 1997). This step is the last one that occurs in plastids. Xanthoxin is exported to the cytoplasm where a two-step reaction via ABA-aldehyde occurs. The first step is catalyzed by a short-chain alcohol dehydrogenase/reductase (SDR) that is encoded by the *AtABA2* (*ABA deficient 2*) gene (Rook et al., 2001; Cheng et al., 2002; Gonzalez-Guzman et al., 2002) and that generates ABA-aldehyde. Then, the ABA-aldehyde oxidase (AAO) with the molybdenum cofactor (MoCo) catalyzes the last step in the biosynthesis pathway – the conversion of ABA-aldehyde into ABA (Seo et al., 2004) (Figure 2A). The appropriate level of active ABA is achieved not only through the biosynthesis and catabolism reactions performed by CYP707A1-4 (cytochrome P450, family 707, subfamily A, polypeptide 1, 2, 3, 4) (Kushiro et al., 2004; Figure 2B), but also by the inactivation of ABA through conjugation and deconjugation. ABA can be inactivated at the C-1 hydroxyl group by different chemical compounds that form various conjugates and that accumulate in vacuoles or in the apoplastic space (Dietz et al., 2000). The most widespread conjugate is ABA glucosyl ester (ABA-GE), which is catalyzed by ABA glucosyltransferase (Boyer and Zeevaart, 1982). Lee et al. (2006) identified the AtBG1 (beta-1,3-glucanase 1) protein that is responsible for the release of ABA from ABA-GE. Their findings showed that ABA deconjugation plays a significant role in providing an ABA pool that allows plants to adjust to changing physiological and environmental conditions (Figure 2C).

The ability of ABA to move long distances allows it to serve as a critical stress messenger. Kuromori et al. (2011) identified the ABA importer – ABCG22 (*Arabidopsis thaliana* ATP-binding cassette G22). The gene encoding this transporter is mainly expressed in the guard cells. In addition, the expulsion of ABA into the intercellular space is mediated by transporters such as ABCG25 (*Arabidopsis thaliana* ATP-binding cassette G25). ABCG25 is expressed primarily in vascular tissues where ABA is synthesized (Kuromori et al., 2010). ABA delivery to the guard cells promotes a cascade of reactions that lead to stomatal closure and that inhibit stomatal opening in order to prevent water loss (Figure 2D).

After ABA is received from ABC transporters by the guard cells, the PYR/PYL/RCAR (pyrabactin-resistance 1/pyrabactin-resistance like/regulatory component of ABA receptor) perceives ABA intracellularly and forms complexes that inhibit clade A of PP2Cs (protein phosphatase 2C), the negative regulators of ABA signaling, such as ABI1 (ABA insensitive 1), ABI2 (ABA insensitive 2), HAB1 (hypersensitive to ABA1) (Ma et al., 2009; Park et al., 2009; Santiago et al., 2009; Nishimura et al., 2010). The inactivation of PP2Cs allows downstream targets to be phosphorylated and activated – Sucrose Non-fermenting 1-Related subfamily 2 protein Kinases (SnRK2) (Fujii and Zhu, 2009; Fujita et al., 2009; Umezawa et al., 2009; Kim et al., 2010). ABA receptors, PP2Cs, and SnRKs form the core of the early ABA signaling cascade (Figure 2E).

### Regulation of stomatal movements during the diurnal cycle – the role of ABA

The ABA mode of action is linked to diurnal stomatal movements. It has been proposed that this link is based on both the molecular connections between ABA and circadian-clock pathways and on ABA biosynthesis and response to light (reviewed in Tallman, 2004). Although several studies have been carried out linking the diurnal cycle with ABA signaling, there is still a need for further research that would clarify this connection. It has been confirmed that the elevated ABA levels in the dark phase of the day are responsible for stomatal closure but, on the other hand, the molecular basis of the sensing CO_2_ molecules by guard cells is still not well understood. This part of investigations still needs confirmation through the use of well-established methods.

**Figure 3:**
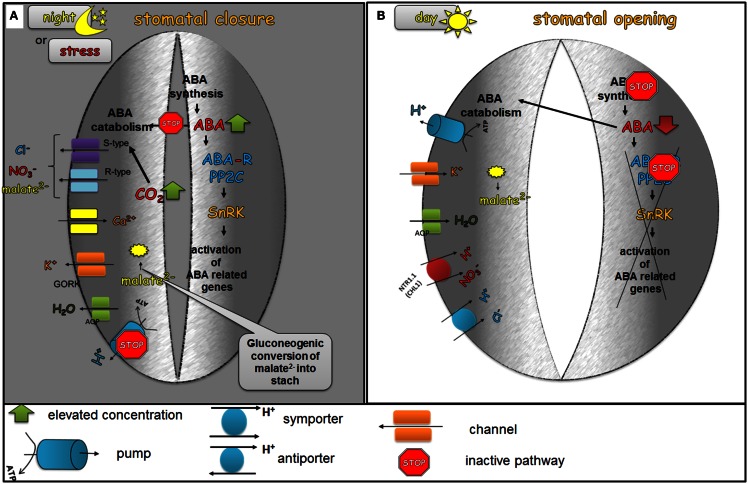
**The role of ABA in the diurnal regulation of stomatal movements**. In the dark phase of the day **(A)**, ABA biosynthesis is favored and at the same time the catabolism of ABA is inhibited. As a result of these processes, elevated levels of ABA are present in the guard cells. ABA activates the efflux of Ca^2+^ from internal stores, the activation of S-type and R-type anion channels leading to the efflux of Cl^−^, malate^2−^, and ${\text{NO}}_{3}^{-}$, the activation of GORK channel, which leads to the efflux of K^+^ and consequently to the closing of stomatal pores. The decreased level of malate^2−^ is also caused by the gluconeogenic conversion of malate into starch. In the dawn **(B)**, the first light promotes ABA catabolism processes and the level of ABA biosynthesis decreases, which leads to a decreased concentration of active ABA in the guard cells. Low endogenous ABA levels no longer inhibit H^+^-ATPase (H^+^-pump), which is then able to extrude H^+^ from the guard cells. At the same time, the accumulation of water and ions, such as K^+^, Cl^−^, malate^2−^ occurs in order to generate the turgor that is needed to keep stomata open.

In darkness, stomata are closed. This is probably caused by an intensive ABA accumulation through the biosynthesis of ABA in the guard cells and the simultaneous import of endogenous ABA from the apoplast to the guard cells using ABA transporters such as ABCG22 (Kuromori et al., 2011), while at the same time, ABA catabolism processes are disfavored. Elevated ABA levels cause stomata closure via the activation of an ABA signaling cascade, the efflux of Ca^2+^ from internal stores, the activation of S-type and R-type anion channels that lead to the efflux of Cl^−^, malate^2−^, and ${\text{NO}}_{3}^{-}$ and the activation of the GORK channel that leads to the efflux of K^+^. During the night, elevated levels of CO_2_ in the leaves were observed due to respiration. It has been proved that CO_2_ has a positive effect on the stomatal closure process. The guard cells probably do not sense CO_2_ molecules but instead ${\text{HCO}}_{3}^{-}$ is synthesized from CO_2_ (Hu et al., 2010), which activates S-type channels and leads to the efflux of Cl^−^, malate^2−^, and ${\text{NO}}_{3}^{-}$ (Xue et al., 2011) (Figure 3A).

At first light, a depletion of endogenous ABA is observed through xanthophyll cycling, the isomerization of ABA precursors and the activation of ABA catabolism enzymes, such as CYP450 (cytochrome P450). The degradation of ABA liberates the guard cells to extrude H^+^ via H^+^-ATPase (H^+^-pump) and accumulate water and ions, such as K^+^, Cl^−^, malate^2−^ in order to generate the turgor needed to keep stomata open. K^+^ uptake is mainly responsible for the rapid increase of the turgor and the opening of stomata during the dawn (Humble and Raschke, 1971; Talbott and Zeiger, 1996). The accumulation of sugars such as glucose, fructose and sucrose has been reported during the light phase of the day (Talbott and Zeiger, 1998). In the midday, ABA is delivered to the apoplast around the guard cells through the xylem transpiration stream and the guard cells are regulated by steady-state ABA concentrations (Figure 3B).

In the evening, ABA biosynthesis outweighs the ABA catabolism in the guard cells, which leads to stomatal closure (for review, see Tallman, 2004).

### ABA on the way to reaching the guard cells under drought stress conditions

Under drought stress conditions, ABA would reach a concentration high enough to cause ion efflux and an inhibition of sugar uptake by the guard cells in the midday, thus reducing the apertures for the rest of the day. Analyses of ABA biosynthesis, catabolism, de/conjugation, and transport have been supported by various studies involving several species and different methods, such as mutant analysis, transcriptomics, proteomics, or immunohistochemical techniques. In order to define the role of ABA in stress response, the action of several components of the pathways mentioned were tested in response to stress. The engagement of such various techniques makes the state of knowledge in the field of ABA biosynthesis, catabolism, de/conjugation, and transport well supported and reliable.

It has been shown that ABA concentrations can increase up to 30-fold in response to drought stress (Outlaw, 2003). Water deficit promotes ABA biosynthesis via the upregulation of a key enzyme – NCED3. A significant increase in NCED transcript levels can be detected within 15–30 min after leaf detachment or dehydration treatment (Qin and Zeevaart, 1999; Thompson et al., 2000), which indicates that the activation of *NCED* genes can be fairly quick. Cheng et al. (2002) reported that the *AtNCED3*, *AtZEP* (*Zeaxanthin epoxidase*), and *AtAAO3* (*ABA-aldehyde oxidase*) genes could be induced in *Arabidopsis* by ABA and studies in rice showed that *OsNCED3* expression was induced by dehydration (Ye et al., 2011). An immunohistochemical analysis, using antibodies raised against AtNCED3, revealed that protein is accumulated in the leaf vascular parenchyma cells in response to drought stress. This was not detected in non-stressed conditions. These data indicate that drought-induced ABA biosynthesis occurs primarily in the vascular parenchyma cells and that vascular-derived ABA might trigger stomatal closure via the transport to the guard cells (Endo et al., 2008). *AtNCED3* expression is upregulated by drought conditions across the species observed and decreases after rehydration.

Drought, like the dark part of a diurnal cycle, also promotes the deconjugation of the ABA-glucose ester (ABA-GE), which is stored in the vacuoles of leaf cells and also circulates in the plant (Xu et al., 2002; Seiler et al., 2011). Both processes, intensive ABA biosynthesis and ABA deconjugation, lead to the accumulation of high levels of biologically active ABA. ABA delivery to the guard cells via ABCG transporters, such as AGCG22 that was mentioned above, promotes a cascade of reactions that lead to stomatal closure and that inhibit stomatal opening in order to prevent water loss (Figure 2).

### ABA triggers changes in ion homeostasis in the guard cells, which leads to stomatal closure under stress

The ABA signaling network that leads to stomatal closure under stress is activated by the perception ABA. This begins a cascade of reactions that leads to the reduced turgor of the guard cells through ABA modulation of ion channel activities, including the regulated efflux of anions and potassium ions and the inhibition of K^+^ import. Recently, the core signalosome of ABA signaling including ABA receptors, phosphatases (PP2Cs), and kinases (SnRK2s) was established (Ma et al., 2009; Park et al., 2009; Santiago et al., 2009; Nishimura et al., 2010). Although its function is clear and confirmed by advanced molecular analysis, there is still a need to explain the impact of single components, such as kinases, on the regulation of the ion channels or the proton pump (e.g., AHA1), which is described below. On the other hand, the interaction between ABA regulated kinases SnRK2s and S-type anion channels, and the potassium inwardly rectifying channels, described below, has been well established and documented.

The inactivation of PP2Cs such as ABI1 and ABI2 by the complex ABA-receptor facilitates the phosphorylation and activation of a downstream target of phosphatases – SnRK2, such as SnRK2.2/D, SnRK2.3/E, and SnRK2.6/OST1/E, which are the key players in the regulation of ABA signaling and abiotic stress response (Fujii and Zhu, 2009; Fujita et al., 2009; Umezawa et al., 2009). Kinases are able to regulate the activity of ion channels and the proton pump. It was shown that ABA inhibits the action of a proton pump such as H^+^-ATPase. The dominant *Arabidopsis* mutant *ost2* (*opened stomata 2*) in *AHA1* (H^+^-ATPase 1 HA1) gene exhibited the constitutive activation of AHA1 H^+^-ATPase, which in turn resulted in an inability to close stomata in response to ABA (Merlot et al., 2007). The molecular mechanism of the inhibition of AHA1 by ABA has not yet been fully elucidated. One of the most direct pieces of evidence of the regulation of H^+^-ATPase by SnRK is the demonstration that specific calcium-stimulated kinase, PSK5 (a member of the SnRK3 kinase family), is able to phosphorylate the closest homolog of AHA1 – AHA2 (H^+^-ATPase 1 HA2) in Ser392 localized in the C-terminus of the AHA2 protein. This reaction prevents the 14-3-3 protein, which is the main activator of AHA2 leading to the inhibition of H+-ATPase action, from binding (Fuglsang et al., 2007).

Sucrose Non-fermenting 1-Related subfamily 2 protein Kinases also regulate S-type anion channels and potassium inwardly rectifying channels such as SLAC1 (slow anion channel-associated 1) and KAT1 (K^+^ channel in *Arabidopsis thaliana*), respectively. The first one is activated by SnRK2, whereas KAT1 is inhibited. *SLAC1* encodes the anion-conducting subunit of an S-type anion channel. In different species, S-type anion channels are activated in the guard cells by ABA, cytosolic Ca^2+^, and phosphorylation events (Schmidt et al., 1995; Pei et al., 1997; Leonhardt et al., 1999; Raschke et al., 2003; Roelfsema et al., 2004; Mori et al., 2006). The *slac1* mutant displayed a strongly impaired response to a range of stomatal closing stimuli such as ABA and Ca^2+^ (Negi et al., 2008; Vahisalu et al., 2008). Increased SLAC1 activity causes an efflux of anions which results in depolarization of the membrane as a consequence of phosphorylation by SnRK. This in turn leads to the loss of K^+^ cations from the cell through the K^+^ efflux channel GORK (guard cell outward-rectifying K^+^), which is activated by depolarization (Jeanguenin et al., 2008). KAT1 is an inward K^+^ channel that allows an influx of K^+^ inside the guard cell when the proton pump drives the PM to a negative potential. When plants encounter drought stress conditions and the ABA level rises, both the proton pumps (as mentioned above) and KAT1 are inactivated by SnRKs. It was shown that the activity of KAT1 is inhibited by an elevation of ABA and cytosolic Ca^2+^ (Schroeder and Hagiwara, 1989; Blatt and Armstrong, 1993; Grabov and Blatt, 1999) via phosphorylation by SnRK, which in turn results in a decreased influx of K^+^ into the guard cells (Hubbard et al., 2010). The loss of K^+^ and anions from the guard cells is accompanied by the efflux of water via aquaporins. Together, these events lead to a reduction of the turgor, which results in stomatal closure in response to ABA as a major signal of drought (Figure 3A).

Abscisic acid activates the Ca^2+^-permeable channels in the PM of the guard cells and triggers an influx of Ca^2+^ into the cytoplasm of the guard cells through the release of the second messenger, inositol-1,4,5-triphosphate (IP_3_), which in turn activates the Ca^2+^ channels that are located in the vacuole and endoplasmic reticulum (Schroeder and Hagiwara, 1990; Hamilton et al., 2000; Krinke et al., 2007; Kwak et al., 2008). Ca^2+^-dependent protein kinases (CDPKs) are activated during drought stress and are able to control stomatal closure in an ABA-dependent manner. After ABA is perceived by a receptor, the action of PP2Cs such as ABI1 are inhibited. ABI1 was identified as a negative regulator of CPK21 (Ca^2+^ dependent protein kinase 21), which like SnRK phosphorylates SLAC1. SLAC1 phosphorylation, in turn, results in the activation of anion and the efflux of K^+^ (Geiger et al., 2010). An increased cytosolic Ca^2+^ level activates the Ca^2+^-dependent pathways that inhibit K^+^ import and trigger the depolarization of the membrane. Mori et al. (2006) identified two calcium-dependent kinases – CPK3 (calcium-dependent protein kinase 3) and CPK6 (calcium-dependent protein kinase 6) as positive regulators of ABA signaling in the guard cells during water stress. Inactivation of both genes led to a reduction in the activation of S-type channels by ABA and Ca^2+^, the impairment of the ABA activation of Ca^2+^ permeable channels and a decreased sensitivity of stomata to ABA. Disruption of the regulatory subunit *RCN1* (*roots curl in NPA*) of the gene encoding PP2A (protein phosphatase 2A) led to a reduction of the ABA activation of anion channels and a decreased sensitivity of stomata to ABA (Kwak et al., 2002, Figure 4).

**Figure 4:**
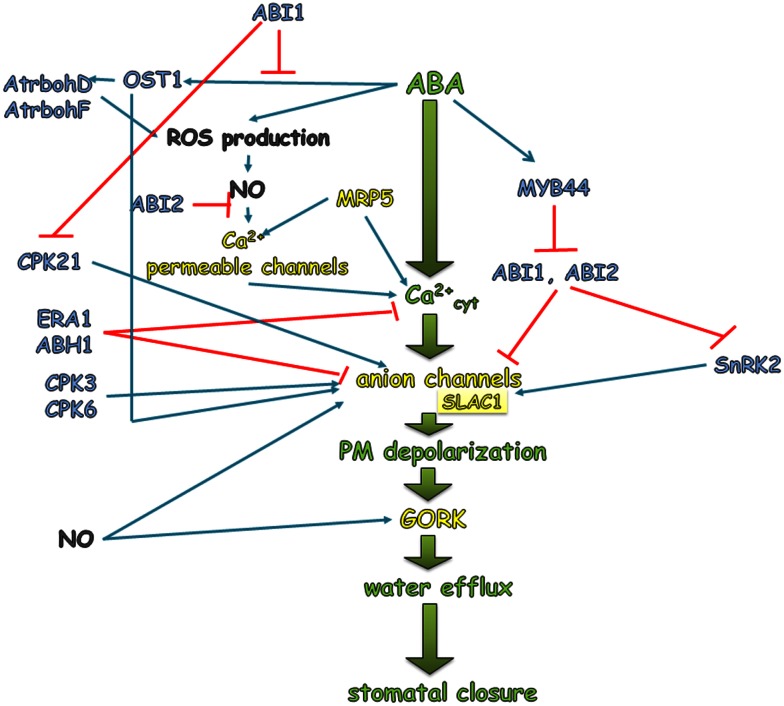
**ABA regulation of stomatal closure during drought stress**. An increased level of endogenous ABA in response to drought activates a signal transduction pathway that involves a sequence of events such as the elevation of the cytosolic Ca^2+^ level, which consequently activates the anion channels (S-type and R-type), which leads to membrane depolarization. The latter activate GORK, which is responsible for extruding K^+^ from the guard cells. Simultaneous with the efflux of K^+^, an efflux of water is observed. Together, these events lead to a decrease in the turgor of the guard cells and to stomatal closure under drought conditions. The sequence of events, which is explained in detail in the main text and presented in green in the figure, is the core of the reactions that are induced or inhibited by different proteins that are activated by ABA. Blue arrows indicate activation, while red blunt ended lines indicate inhibition.

Another protein involved in ABA signaling in stomata is GPA1. GPA1 is a positive regulator in the ABA-mediated inhibition of stomatal opening. *Arabidopsis* plants lacking GPA1 (Gα subunit of G protein) showed a reduction in the inhibition of inward K^+^ currents and a reduced guard cell ABA-insensitivity in stomatal opening (Wang et al., 2001). The mutants *era1* (*enhanced response to ABA1*) and *abh1* (*ABA hypersensitive 1*), which are deficient in a farnesyl transferase subunit and RNA cap-binding protein, respectively, are ABA hypersensitive and showed an enhanced ABA activation of S-type channels (Pei et al., 1998; Schroeder et al., 2001a; Hugouvieux et al., 2002; Figure 4). However, the exact molecular role of ERA1 or ABH1 in stomatal regulation should be clarified in future research.

During stomatal closure, slow vacuolar (SV) channels activated by cytosolic Ca^2+^ contribute to the release of Ca^2+^ from vacuoles. SV channels were shown to be calcium permeable and it was suggested that they facilitate a brief transient efflux of cations, including Ca^2+^, from vacuoles (Ward and Schroeder, 1994).

Several of the genes involved in the processes described above and more are presented in Table 1 together with a description of mutant phenotypes.

**Table 1:** **Selected genes involved in the regulation of stomatal movement under stress**.

Gene	Description	Mutant	Phenotype	Reference
*ABH1*	Encodes a nuclear cap-binding protein that forms a heterodimeric complex with CBP20 and is involved in ABA signaling	*abh1*	ABA hypersensitive, shows enhanced ABA activation of S-type channels	Schroeder et al. (2001a), Hugouvieux et al. (2002)
*ABI1*	Encodes the protein phosphatase 2C involved in abscisic acid (ABA) signal transduction. Negative regulator of stomatal closure promoted by ABA	*abi1*	Improper stomatal regulation leading to increased transpiration	Parcy and Giraudat (1997)
*ABI2*	Encodes the protein phosphatase 2C involved in abscisic acid (ABA) signal transduction. Negative regulator of stomatal closure promoted by ABA	*abi2*	Improper stomatal regulation leading to increased transpiration	Pei et al. (1997)
*AHA1*	Encodes a plasma membrane proton ATPase	*ost2*	Constitutively activated H+-ATPases, insensitivity to ABA persisted stomatal opening and a reduced ability to close stomata in response to drought	Merlot et al. (2007)
*ALMT12*	Encodes an anion transporter involved in stomatal closure	*almt12*	Impaired stomatal closure in response to ABA, darkness and CO_2_	Meyer et al. (2010)
*AtrbohD*	Encodes the NADPH/respiratory burst oxidase protein D (RbohD).Interacts with AtrbohF	*atrbohd*	Impaired stomatal closure in response to ABA	Kwak et al. (2003)
*AtrbohF*	Encodes the NADPH/respiratory burst oxidase protein F (RbohF). Interacts with AtrbohD	*atrbohf*	Impaired stomatal closure in response to ABA	Kwak et al. (2003)
*COI*	Encodes a protein containing Leu-rich repeats and a degenerate F-box motif	*coi*	Disrupted activation of S-type anion channels	Munemasa et al. (2007, 2011)
*CPK10*	Encodes the calcium-dependent protein kinase whose gene expression is induced by dehydration and high salt	*cpk10*	Sensitive to drought, impaired stomatal closure	Zou et al. (2010)
*CPK21*	Encodes a member of the calcium-dependent protein kinase	*cpk21*	Tolerant to osmotic and drought stress	Franz et al. (2010)
*CPK3*	Encodes the calcium-dependent protein kinase 3 (CPK3), a member of the *Arabidopsis* CDPK gene family. CPK3 is expressed in both guard cells and mesophyll cells. Functions in guard cell ion channel regulation	*cpk3*	Reduction in ABA and Ca^2+^ activation of S-type channels, impaired ABA activation of Ca^2+^ permeable channels, decreased ABA sensitivity to stomatal closure	Mori et al. (2006)
*CPK6*	Encodes the calcium-dependent protein kinase 3 (CPK3), a member of the *Arabidopsis* CDPK gene family. CPK3 is expressed in both guard cells and mesophyll cells. Functions in guard cell ion channel regulation	*cpk6*	Reduction in ABA and Ca^2+^ activation of S-type channels, impaired ABA activation of Ca^2+^ permeable channels, decreased ABA sensitivity to stomatal closure	Mori et al. (2006), Munemasa et al. (2011)
*ERA1*	Encodes a beta subunit of farnesyl-trans-transferase, which is involved in meristem organization and the ABA-mediated signal transduction pathway. Mutant phenotypes were observed in meristem organization and response to abscisic acid and drought	*era1*	ABA hypersensitive and showed enhanced ABA activation of S-type channels	Pei et al. (1998)
*ERF7*	Encodes a member of the ERF (ethylene response factor) subfamily B-1 of the ERF/AP2 transcription factor family (ATERF-7). The protein contains one AP2 domain. Phosphorylated by PKS3 *in vitro*. Involved in ABA-mediated responses	*erf7*	Increased sensitivity of stomata to ABA compared to the wild-type, enhanced drought tolerance	Song et al. (2005)
*GORK*	Encodes a guard cell outward potassium channel. Belongs to the Shaker family K + channel	*gork*	Impaired stomatal closure	Hosy et al. (2003)
*GPA1*	Encodes an alpha subunit of a heterotrimeric GTP-binding protein. GPA1 is a positive regulator in ABA-mediated inhibition of stomatal opening	*gpa1*	Reduction in the inhibition of inward K^+^ currents, reduced guard cell ABA-insensitivity in stomatal opening	Wang et al. (2001)
*KAT1*	Encodes a potassium channel protein (KAT1)	*kat1*	No impairment of stomatal action, but potassium currents were altered	Szyroki et al. (2001)
*MRP5*	Encodes a high-affinity inositol hexakisphosphate transporter that plays a role in guard cell signaling and phytate storage. It is a member of the MRP subfamily/ABC transporter subfamily C	*mrp5*	Impaired ABA regulation of Ca^2+^ permeable channels, defects in S-type channel regulation	Suh et al. (2007)
*MYB15*	Encodes a member of the R2R3 factor gene family	*35S:myb15*	More sensitive to ABA-induced stomatal closure, improved drought tolerance	Ding et al. (2009)
*MYB44*	Encodes a member of the R2R3 factor MYB gene family involved in mediating plant responses to a variety of abiotic stimuli	*35S:myb44*	More drought tolerant	Jung et al. (2007)
*MYB60*	Encodes a putative transcription factor of the R2R3-MYB gene family. Transcript increases under conditions that promote stomatal opening (white and blue light) and decreases under conditions that trigger stomatal closure (ABA, desiccation, darkness) with the exception of elevated CO_2_. Expressed exclusively in the guard cells of all tissues. It is required for light-induced opening of stomata	*myb60*	Reduced stomatal aperture which helps to limit water loss during a drought	Cominelli et al. (2005)
*MYB61*	Encodes the putative transcription factor. Expressed in guard cells, plays a role in the regulation of stomatal pore size	*myb61*	Larger stomatal pores than the wild-type	Liang et al. (2005)
*NFYA5*	Encodes a member of the CCAAT-binding transcription factor (CBF-B/NF-YA) family. Expression is upregulated in response to ABA and drought	*nfya5*	Hypersensitive to drought because their stomata are more open than the wild-type	Li et al. (2008)
*NPX1*	Encodes NPX1 (Nuclear Protein X1), a nuclear factor that regulates abscisic acid responses	*npx1*	Stomata were more closed than the wild-type in response to ABA and were more drought tolerant	Kim et al. (2009)
*NRT1.1 (CHL1)*	Encodes NRT1.1 (CHL1), a dual-affinity nitrate transporter. The protein is expressed in guard cells and functions in stomatal opening	*nrt1.1* (*chl*)	Lower transpiration rate and tolerant to drought	Guo et al. (2003)
*PUB18*	Encodes a protein containing a UND, a U-box and an ARM domain	*pub18*	Hypersensitive to ABA-mediated stomatal closure	Seo et al. (2012)
*PUB19*	Encodes PUB19, a plant U-box armadillo repeat protein. Involved in the salt inhibition of germination together with PUB18	*pub19*	Hypersensitive to ABA-mediated stomatal closure	Liu et al. (2011)
*SLAC1*	Encodes a membrane protein with 10 predicted transmembrane helices. SLAC1 is a multispanning membrane protein that is expressed predominantly in the guard cells that play a role in regulating cellular ion homeostasis and S-type anion currents. SLAC1 is important for normal stomatal closure in response to a variety of signals including elevated CO_2_, ozone, ABA, darkness and humidity. SLAC1:GFP localizes to the plasma membrane	*slac1*	Reduced stomatal closure response to ABA, CO_2_, Ca^2+^ and ozone treatments	Vahisalu et al. (2008)

*Pink indicates genes that encode the negative regulators of ABA signaling, blue indicates genes that encode ion channels, pump, and transporters localized in the plasma membrane of guard cells, green indicates genes that encode the Ca^2+^-dependent protein kinases involved in the regulation of stomatal movements, brown indicates genes that encode the transcription factors involved in the regulation of stomatal movements*.

### NO and ROS in response to drought stress and ABA

The guard cells generate reactive oxygen species (ROS) such as hydrogen peroxide (H_2_O_2_) and nitric oxide (NO) in response to ABA (Pei et al., 2000; Zhang et al., 2001). Exogenous H_2_O_2_ activates permeable Ca^2+^ channels in the PM of *Arabidopsis* guard cells and inhibits inward K^+^ channels (Zhang et al., 2001; Kohler et al., 2003; Kwak et al., 2003). Taking into account the fact that ROS and NO signaling is not yet fully understood, there is a need for further analysis in order to elucidate their function, for example, the role of Ca^2+^ in ROS and NO action in guard cells should be clarified.

Reactive oxygen species production in *Arabidopsis* guard cells is mediated by two subunits of NADPH oxidase – AtrbohD (*Arabidopsis thaliana* respiratory burst oxidase homolog D) and AtrbohF (*Arabidopsis thaliana* respiratory burst oxidase homolog F). The significance of ROS involvement in stomatal closure was revealed by an analysis of the *atrbohD*/*atrbohF* double mutant, which showed impaired stomatal closure in response to ABA (Kwak et al., 2003). The protein, OST1 (open stomata1), displays dominant kinase activity during drought stress response and is able to activate NADPH oxidase (Sirichandra et al., 2009). Mutants in *OST1* showed a wilty phenotype in water deficit conditions because of the impairment of stomatal closure and ROS production (Mustilli et al., 2002; Yoshida et al., 2006; Figure 4).

Another crucial factor for stomatal closure is NO, which is generated in response to ABA (Neill et al., 2002, 2008). Exogenously applied NO donors triggered stomatal closure, whereas the application of an NO scavenger inhibited ABA-induced stomatal closure (Neill et al., 2002; Figure 4).

There is some evidence that both H_2_O_2_ and NO actions in the guard cells require calcium. In addition, H_2_O_2_ inhibits K^+^ channel activity, induces cytosolic alkalization in the guard cells and promotes NO signaling in response to ABA (Zhang et al., 2001; Kohler et al., 2003; Wang and Song, 2008). Conversely, NO neither stimulates H_2_O_2_ synthesis nor does it require H_2_O_2_ for its action (Bright et al., 2006).

## The Second Violin in the Concert of Stomatal Closure – The Role of Jasmonates in the Regulation of Stomatal Movement

Jasmonates are lipid-derived phytohormones that are involved in the regulation of vegetative and reproductive growth and the defense response against abiotic stress (Katsir et al., 2008). JA biosynthesis is induced by stress conditions (Wasternack, 2007) and many genes related to JA signaling are regulated by drought stress (Huang et al., 2008). The positive role of JA in the regulation of stomatal closure was observed in many studies (Gehring et al., 1997; Suhita et al., 2003, 2004; Munemasa et al., 2007). Similar to the ABA signaling pathway, JA signaling has been under intense investigation, particularly in relation to stress response. With the progress in research, many new components and their roles in JA-mediated stress response will be identified. Although the interaction between ABA and JA signaling pathways in stomata function has been established, there is still a need for further investigation and identification of the nodes linking these two signaling pathways, such as CPK6, which is described below.

**Figure 5:**
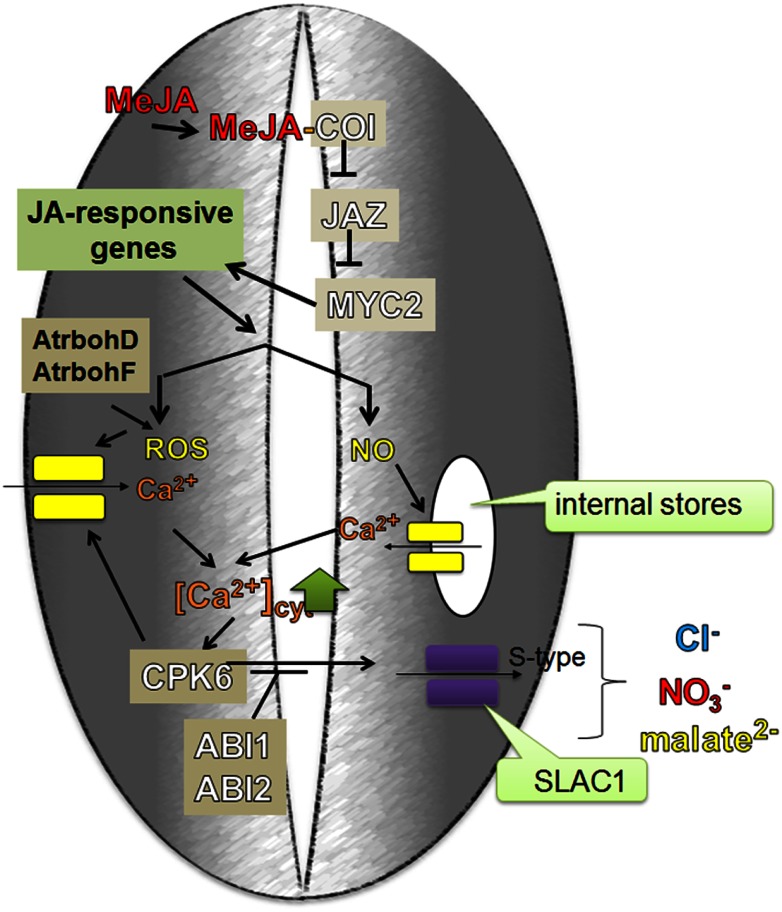
**Me-JA regulated stomatal closure during drought stress**. MeJA, before it can be bound by a receptor in the plant cell, is converted into a biologically active form (+)-7-iso-Jasmonoyl-l-isoleucine (JA-Ile). JA-Ile is then bound by the receptor ^SCF^COI complex that contains the coronatine insensitive1 (COI1) F-box protein. This interaction leads to the JAZ degradation which is negative regulator of MYC2. Inactive JAZ is not able to repress MYC2 function which in turn activates JA-responsive genes. MeJA induces the formation of ROS and NO, which activate the efflux of Ca^2+^ from internal stores and the influx from the apoplast by channels in plasma membrane. CPK6 acts downstream of NO and ROS signaling and therefore may be the target of an NO-stimulated influx of Ca^2+^ into the cytoplasm. As a feedback loop, MeJA-induced influx of Ca^2+^ into the cytoplasm activates CPK6, which in turn is able to activate the S-type anion channel – SLAC1, which then leads to the MeJA-stimulated stomatal closure.

When JA or methyl JA (MeJA) are applied exogenously to plants, they are converted into a biologically active form (+)-7-iso-Jasmonoyl-l-isoleucine (JA-Ile). JA-Ile is then bound by the receptor ^SCF^COI complex that contains the coronatine insensitive1 (COI1) F-box protein (Fonseca et al., 2009; Sheard et al., 2010). This interaction leads to the degradation of the repressor protein, JAZ (Jasmonate ZIM-domain), by the 26S proteasome and as a result, to the activation of distinct JA response genes by MYC2 (MYC domain transcription factor 2) (Chini et al., 2007; Thines et al., 2007; Fernández-Calvo et al., 2011). In the absence of JA, JAZ inhibits MYC2, which is then unable to activate the transcription of JA-inducible genes (Figure 5).

**Figure 6:**
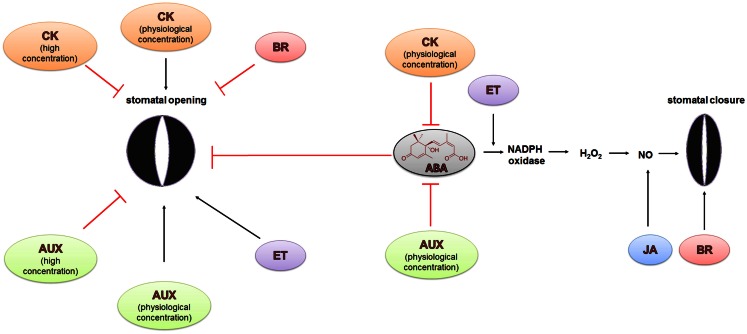
**Hormonal crosstalk in the regulation of stomatal closure and opening during water stress**. The regulation of stomatal opening and closure is not only regulated by ABA, whose role is dominant, but also by other phytohormones. Jasmonates (JA) and brassinosteroids (BR) induce stomatal closure and inhibit stomatal opening under drought conditions, whereas the role of other hormones is ambiguous. Cytokinins (CK) and auxins (AUX) in low physiological concentrations promote stomatal opening while in high concentrations, they are able to inhibit this process. The role of ethylene (ET) is the most curious. It can stimulate the closing and opening of the stomata. The details are described in the text.

Munemasa et al. (2011) identified CPK6 (Ca^2+^ dependent protein kinase 6), which had previously been studied by Mori et al. (2006) in regards to ABA signaling, as a positive regulator of MeJA signaling in the guard cells. CDPKs function as important cytosolic Ca^2+^ sensors in various plant physiological processes. Four kinases are involved in ABA signaling in *Arabidopsis* guard cells: CPK3, CPK6, CPK4, and CPK11; however, only mutations in the *CPK6* impaired MeJA-induced stomatal closure (Munemasa et al., 2011). Like ABA, MeJA activates S-type anion channels. In *coi1* (*coronatine insensitive 1*) and *cpk6* mutants, the activation of S-type anion channels was disrupted (Munemasa et al., 2007, 2011). Geiger et al. (2010) showed a direct interaction between CPK6 and the SLAC1 – S-type anion channel. The activation of SLAC1 by CPK6 was inhibited by the PP2Cs, ABI1, and ABI2, since *abi1* and *abi2* mutants exhibited insensitivity of stomata to MeJA, which leads to the inability of stomatal closure in response to MeJA (Figure 6).

The formation of ROS and NO in the guard cells is not only induced by ABA and ethylene but also by MeJA. It has been showed that both ROS and NO levels were decreased in MeJA-insensitive mutants (Munemasa et al., 2007). Suhita et al. (2004) showed that a disruption of both genes encoding NADPH oxidase, *AtrbohD* and *AtrbohF*, results in the impairment of MeJA-induced stomatal closure and ROS production. However, in the *cpk6* *Arabidopsis* mutant, ABA- and MeJA-mediated the production of ROS and NO was not reduced. CPK6 acts downstream of NO and ROS signaling and therefore may be a target of the NO-stimulated influx of Ca^2+^ into the cytoplasm. As a feedback loop, MeJA-induced influx of Ca^2+^ into the cytoplasm activates CPK6, which in turn is able to activate the S-type anion channel – SLAC1 (Figure 5). This property of CPK6 makes it a node between the NO, ROS, ethylene and JA signaling pathways in ABA-induced stomatal closure (Munemasa et al., 2011; Figure 6).

Jasmonates interacts with the ABA pathway by increasing the influx of Ca^2+^, which stimulates CDPK and the resulting cascade in order to close stomata. Munemasa et al. (2007) reported that ABA or MeJA treatment triggers a reduction in the stomatal aperture within 10 min. MeJA-induced Ca^2+^ levels were significantly lowered and stomatal closure was impaired when ABA biosynthesis inhibitors were added or when ABA-deficient mutants were studied. This suggests that jasmonate-induced changes in stomatal movements require endogenous ABA. In order to clarify this hypothesis, Hossain et al. (2011) examined the effect of 0.1 μM of ABA on MeJA-induced stomatal closure in *aba 2-2* (*ABA deficient 2-2*) mutants related to ABA biosynthesis. In the wild-type, 0.1 μM of ABA did not significantly induce either stomatal closure or Ca^2+^ oscillations. The authors did not observe stomatal closure in *aba2-2* when MeJA was applied without ABA, while in the presence of 0.1 μM ABA, MeJA induced stomatal closure.

## When ABA Meets Ethylene

Ethylene is a gaseous phytohormone that is involved in the regulation of numerous plant processes such as seed germination, root-hair growth, leaf and flower senescence and abscission, fruit ripening, nodulation, and plant responses to stresses (Bleecker and Kende, 2000). It has been observed that ethylene can influence stomatal response via crosstalk with ABA; however, reports on its effect have been contradictory. Ethylene has been linked to the promotion of both stomatal closure (Pallas and Kays, 1982) and stomatal opening (Madhavan et al., 1983; Levitt et al., 1987; Merritt et al., 2001; Figure 6). These contradictory effects need to be verified. One possible reason could be related to the methods used for stomatal observation that use detached leaves. Experiments with detached leaves do not always reflect the real response to stress or other applied factors in plants.

Tanaka et al. (2005) showed that *Arabidopsis* plants exposed to gaseous ethylene first did not close their stomata after the application of ABA. This was clear evidence that ethylene repressed ABA action in stomatal closure. In a drought stressed *eto1* (*ethylene overproducer 1*) mutant, stomata closed more slowly and were less sensitive to ABA than in the drought-treated wild type (Tanaka et al., 2005). In order to elucidate the interaction between ethylene and ABA during stomatal response, epidermal peels from the wild-type and *eto1* were treated with ABA, ethylene, and both phytohormones. When ethylene was applied independently of ABA, it induced H_2_O_2_ synthesis within 30 min of the treatment. When ethylene was applied to the ABA-pretreated wild-type epidermal peels, an inhibition of stomatal closure was observed (Tanaka et al., 2005). Desikan et al. (2006) proved that ethylene-mediated stomatal closure is dependent on the H_2_O_2_ that is generated by NADPH oxidase. As was discussed above, H_2_O_2_ is one of the major molecules in ABA-induced stomatal closure.

There have been some studies that revealed both increased and decreased ethylene production in response to drought stress. However, most of them described experiments with detached leaves, which may not reflect the response of intact plants under drought conditions (Morgan et al., 1990; Abeles et al., 1992). Generally, elevated ABA concentrations limit the production of ethylene; and therefore a dramatic increase of ABA concentration during water stress probably causes a reduction in the production of ethylene (Sharp, 2002). The physiological mechanism of ethylene inhibition of the ABA-mediated stomatal closure may be related to the function of ethylene as a factor that ensures a minimum carbon dioxide supply for photosynthesis by keeping stomata half-opened under the stress conditions (Leung and Giraudat, 1998; Tanaka et al., 2005).

## Auxins and Cytokinins – Ambigous Participation in Stomatal Movements

Auxins and cytokinins are major phytohormones that are involved in processes related to plant growth and development such as cell division, growth and organogenesis, vascular differentiation, lateral root initiation as well as gravi- and phototropism (Berleth and Sachs, 2001). Auxins typically play a positive role in stomatal opening but high concentrations of auxin can inhibit stomatal opening (Lohse and Hedrich, 1992; Figure 6). Auxins stimulate the PM H^+^-ATPase in the guard cells. Proton efflux leads to the hyperpolarization of the membrane and results in an uptake of K^+^. Low auxin concentrations activate inward K^+^ channels leading to stomatal opening, whereas high auxin level promotes outward K^+^ channels, while simultaneously inhibiting inward K^+^ channels, which results in stomatal closure (Lohse and Hedrich, 1992; Blatt and Thiel, 1994).

The impact of cytokinins on stomatal movements is also ambiguous. It has been shown that an increased cytokinin concentration in xylem sap promotes stomatal opening and decreases sensitivity to ABA. Drought stress inhibits the synthesis of cytokinins in roots and its transport to shoots, which in turn results in stomatal closure (Pospísilova, 2003; Pustovoitova et al., 2003). However, stomatal response to exogenously applied cytokinins depends on the concentration and cytokinin species (Figure 6). Generally, exogenous cytokinins and auxins can inhibit ABA-induced stomatal closure in diverse species (Stoll et al., 2000; Tanaka et al., 2006).

## Brassinosteroids Play in the Same Team with ABA

Brassinosteroids (BR) are polyhydroxylated steroidal phytohormones that are involved in seed germination, stem elongation, vascular differentiation, and fruit ripening (Clouse and Sasse, 1998; Steber and McCourt, 2001; Symons et al., 2006). It has been shown that epibrassinolide (eBL) promotes stomatal closure and inhibits stomatal opening in epidermal peels of *Vicia faba* through the negative regulation of the inwardly rectifying K^+^ channels that are responsible for the uptake of K^+^ during stomatal opening (Haubrick et al., 2006; Figure 6). eBL is able to activate the transcription of drought-inducible genes in *Arabidopsis*, such as *RD29A* (*response to drought 29A*), *ERD10* (*early response to drought 10*), and *RD22* (*rehydration responsive 22*) (Kagale et al., 2007). Together, these results suggest that there is an interaction between BR and ABA in drought response that is related to stomatal closure.

## The-State-of-Art and Weak Points in Our Understanding of Stomatal Movements

Stomata are epidermal pores on a plant’s surface that are essential for the control of water balance in plants. Many factors that are responsible for the regulation of stomatal movements have been already identified, such as components of ABA and other phytohormone signaling pathways. The most important, and one that is supported by well-documented studies, is the interaction between ABA (when taking into account its biosynthesis, catabolism, de/conjugation, and core signalosome) and the pumps and ion channels in the guard cell PM, in the regulation of stomatal movements under the stress.

However, further analyses of the networks of protein interactions, the co-expression of genes, metabolic factors, etc. should provide new insights into the key regulators of drought response in relation to guard cell movements. Taking into account that phytohormone pathways are still under intensive investigations and there are still many gaps to be elucidated, many of the already established interactions may be changed as further progress in research is achieved.

There are ambiguous reports in regards to the role of some phytohormones, such as ethylene, auxins, or cytokinins, in the regulation of stomatal movement that need to be clarified. In addition, the interaction between the diurnal cycle and ABA pathway should be further investigated in order to achieve a full understanding of this process.

There are some points that should be highlighted as a possible cause of the ambiguous reports related to the action of the regulators of stomatal movements. The first of these is the technique that is used to observe the stomata. Most analyses of stomata under stress are based on stomatal aperture observations. Some studies rely on stomata replicas from plants treated with stress and control, and observed under the light microscopy. This method is simple and inexpensive but generates problems due to the type of material used for the replicas. The accuracy and precision in the determination of stomatal aperture width is limited by the resolution of the standard light microscope. In contrast, scanning microscopy (SEM) offers high resolution images of stomata but requires expensive equipment and is not suitable for collecting large numbers of probes (Lawson et al., 1998). Recently, a popular technique in stomatal observations is confocal microscopy (Cañamero et al., 2006). As long as a proper technique that is not controversial in regards to its influence on stomatal response is not applied, all aperture measurements will be under discussion.

Another crucial problem is that most reports describe experiments with detached leaves, which may not reflect the response of intact plants under drought conditions (Morgan et al., 1990; Abeles et al., 1992; Dodd, 2012). Franks and Farquhar (2007) addressed the problem of data integration in stomatal research. They pointed out the lack of the integration of mechanical and quantitative physical information about guard cells and adjacent cells in model of stomatal function. Such integration of data should allow gas-exchange regulation to be better described and predicted. As long as guard cells are considered as a model without their surroundings, the results obtained may not be relevant. Another problem noted by Franks and Farquhar (2007) is that research on the impact of various environmental factors on the stomatal regulation and stomatal density should be performed on and compared among several species, not only one. This would allow a full picture of a broad morphological and evolutionary spectrum of possibilities of stomata development, density, and movement regulation in response to stresses to be obtained.

Summarizing, there are still many questions about the techniques used for evaluating the stomatal response to stress. Further development of proper methods will bring us closer to a fuller and more relevant understanding of stomatal action. The great progress in molecular biology studies enable insights into the signaling pathways, identification of new components, and interactions between them to be gained.

## Conflict of Interest Statement

The authors declare that the research was conducted in the absence of any commercial or financial relationships that could be construed as a potential conflict of interest.
